# Racial Disparities in the Diagnosis and Management Between Secondary Care Ethnic Minority and White British Patients With Irritable Bowel Syndrome

**DOI:** 10.1111/nmo.70272

**Published:** 2026-03-10

**Authors:** Amalie Newman‐Booth, Emma Fairhurst, Dipesh H. Vasant

**Affiliations:** ^1^ University of Manchester Manchester UK; ^2^ Neurogastroenterology Unit, Wythenshawe Hospital Manchester University, NHS Foundation Trust Manchester UK

**Keywords:** disorders of gut‐brain interaction, irritable bowel syndrome, racial disparities

## Abstract

**Background:**

Irritable bowel syndrome (IBS) is a highly prevalent disorder of gut‐brain interaction best understood within a biopsychosocial framework. Recent studies in other healthcare systems have suggested racial disparities in the management of IBS.

**Aims:**

We aimed to investigate racial disparities in the diagnosis and management of IBS including adherence to national guidelines between White British and Ethnic minority patients with IBS in a UK secondary care setting.

**Methods:**

Consecutive Ethnic minority patients (*N* = 68) with a coded secondary care diagnosis of IBS at a gastroenterology department in a large UK teaching hospital were identified from electronic health records. Data on diagnostic pathways and access to treatments and adherence to national guidelines were compared statistically with an equal number of age and gender matched white British controls (*N* = 68).

**Results:**

Compared to age and gender matched White British controls, Ethnic Minority patients saw more clinicians (*p* = 0.012) and required more outpatient appointments to make an IBS diagnosis (*p* = 0.007). There were disparities identified in the approach to treatment, with ethnic minority patients less likely to be recommended second‐line pharmacological treatment (*p* = 0.004) and Brain‐Gut Behavioral Therapies (*p* = 0.005) compared to their White British counterparts. Across both groups, adherence to national guidelines in the diagnostic approach and treatment for IBS was low, with most patients not being recommended second‐line medical, dietary, or behavioral treatment for their IBS.

**Conclusions:**

These data suggest that the management of IBS in secondary care in the UK has not kept pace with advances in evidence‐based treatments and updated guidelines. Moreover, racial disparities, whether influenced by clinicians or patients, were seen between the two ethnic groups regarding the diagnosis and management of IBS. Further studies are necessary to determine the barriers contributing to these disparities, to influence future interventions and clinical training to address them.

## Key Points

- Compared to age and gender matched White British controls, ethnic minority patients with IBS in secondary care see on average more clinicians and require more clinic appointments to receive an IBS diagnosis.
- Ethnic minority patients are also less likely to be recommended second‐line pharmacological and brain‐gut behavioural therapies than White British patients with IBS in secondary care.
- Overall, regardless of ethnic group, adherence to IBS diagnosis and management guidelines in secondary care appears to be poor.

## Graphical abstract

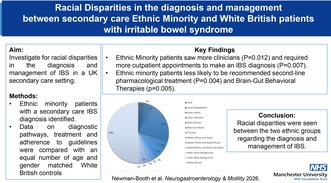

## 1 Introduction

Irritable bowel syndrome (IBS) is a highly prevalent, multifactorial bowel disorder of gut brain interaction (DGBI) with an estimated global prevalence of 4.1% [1]. Recent international studies have demonstrated slight differences in the prevalence and severity of IBS between countries, possibly influenced by sociocultural factors including dietary patterns [1] IBS is characterized by an altered bowel habit and pain associated with defaecation [2, 3], and causes considerable impairment in quality of life [4].

It is widely accepted that IBS results from complex interactions between environmental, biological, and psychosocial factors. Factors such as ethnicity, cultural differences, and stigma surrounding a DGBI can influence a patient's healthcare utilization and reveal racial disparities in IBS. Widespread ethnic inequalities have been found across the National Health service (NHS) in the UK, with ethnic minorities being negatively affected by poor experiences and outcomes when seeking help, which could contribute to a delay in diagnosis and treatments [5]. Alongside racial disparities in IBS, stigma surrounding DGBI, which can be embedded in ethnic culture, can undermine quality of care and contribute to reduced health‐seeking behaviors, adherence, and self‐efficacy [6, 7].

Guidelines from the British Society of Gastroenterology (BSG) assert that the diagnosis of IBS can be made in primary care by general practitioners, based on a history of characteristic symptoms and absence of ‘red flag’ symptoms. Despite this, patients with IBS symptoms are often referred to secondary care without a confident diagnosis [8]. Furthermore, studies from North America highlighted differences in the diagnostic approaches taken towards IBS in ethnic minorities compared with a white control group [9].

In the British Society of Gastroenterology (BSG) guidelines, management of IBS is clearly set out in a step‐by‐step approach from dietary modifications, first line, second line, neuromodulator, and Brain‐Gut Behavioral Treatments [8]. However, there is evidence that a lack of medical training and familiarity with a DGBI may lead to a delayed diagnosis and incorrect management, as physicians are hesitant to treat IBS, resulting in more referrals to secondary care [10, 11].

In this context, we aimed to investigate racial disparities in IBS by comparing the diagnosis and management of IBS according to BSG guidelines between ethnic minorities and white British controls. Guided by patterns in prior literature and similar studies conducted in the USA, we hypothesized that patients from an ethnic minority would undergo on average more investigations and would wait longer for a confident IBS diagnosis [9]. We hypothesized that they would have access to fewer treatments and be more likely to be discharged from secondary care without getting symptomatic relief from their IBS. Identifying ethnic disparities in IBS care could guide clinicians in adapting their practices, ensuring equitable and effective management of the condition.

## 2 Methods and Materials

### 2.1 Study Design and Patient Population

We conducted a retrospective service evaluation of healthcare records of patients seen by a secondary care clinician with an outpatient clinic encounter coded diagnosis of irritable bowel syndrome (IBS) in the gastroenterology department at a large UK Teaching Hospital Trust (Manchester University NHS Foundation Trust) encompassing four hospitals (Manchester Royal Infirmary, Wythenshawe Hospital, North Manchester General Hospital, and Trafford General Hospital) between September 2022 and May 2025.

Patients were identified using the SlicerDicer tool on HIVE (EPIC), an electronic healthcare records system which came to use in September 2022. We identified these patients from a search of Systematized Nomenclature of Medicine Clinical Terms (SNOMED CT), using the search terms ‘irritable bowel syndrome’, ‘irritable bowel syndrome with predominant constipation’, ‘irritable bowel syndrome with predominant diarrhoea’, and ‘irritable bowel syndrome with alternating bowel habit’. We then excluded patients who received diagnoses in the emergency department, leaving us with a group of patients seen by a gastroenterology clinician in secondary care for their IBS. We then grouped these patients by their self‐identifying ethnic group, such as “White British” or “Asian Pakistani”. Patients diagnosed and coded with IBS in a clinical encounter with the regional tertiary IBS/neurogastroenterology clinic were excluded from this study of secondary care patients. However, where patients with a secondary care encounter IBS diagnosis were referred for tertiary IBS input, this was recorded.

**FIGURE 1:**
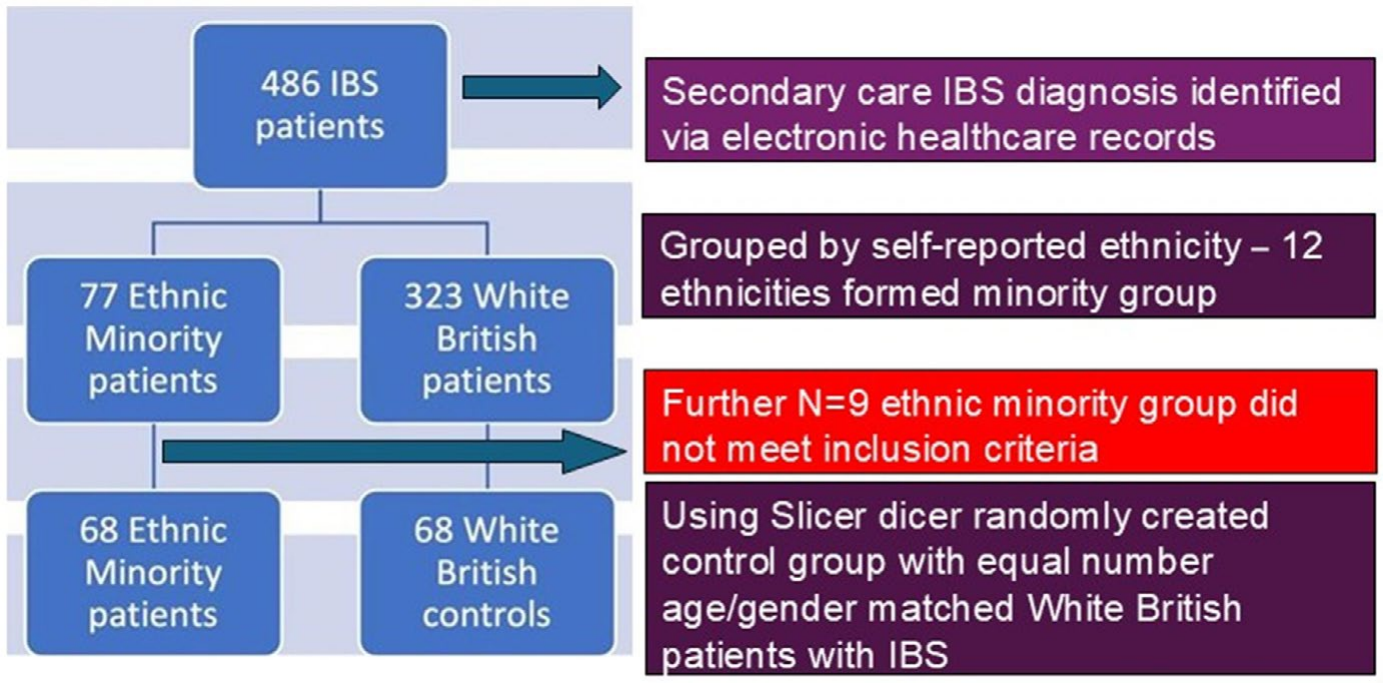
Summary of study design and patient population.

The search identified 486 patients with a secondary care clinician encounter diagnosis of IBS. This included 323 white British patients, and 77 patients with IBS across 12 ethnic minority groups. To enable us to compare the care of each cohort minimizing bias, we created a control group of White British patients, who were randomly gender and age (±2 years) matched to the ethnic minority group. When collecting patients' data using EPIC (electronic healthcare database), those who did not meet the criteria of having a definitive encounter diagnosis of IBS, or who did not see a secondary clinician for their IBS specifically were excluded. This removed 20 patients, 9 White Ethnic minorities and 11 White British patients, leaving us with 68 patients in each cohort that met all the criteria (Figure 1).

### 2.2 Data Collection

Retrospectively, data were collected on patient demographics including age, gender, religion, and ethnicity. Data were also collected on healthcare utilization data including IBS diagnostic and management pathways compared to national guidelines (2021 BSG guidelines) [8].

Data collected included IBS subtypes; relevant comorbidities; whether they had a primary care IBS diagnosis; if they were diagnosed with IBS at their initial secondary encounter, and how many clinicians and clinical appointments they had before receiving their IBS diagnosis. We also recorded the number of investigations performed by primary and secondary care providers, including non‐invasive tests such as fecal calprotectin, coeliac serology, fecal elastase, fecal immunochemical, SIBO breath test and lactose intolerance breath test. We recorded whether patients had procedures such as colonoscopy, flexible sigmoidoscopy, colonic biopsies, duodenal biopsies, OGD, CT scan abdomen, ultrasound small bowel, MRI small bowel, and SeHCAT test, and where multiple, how many they had. Data was collected on the time interval from the patient's initial encounter in secondary care to IBS diagnosis, excluding those with a primary care diagnosis. The total number of invasive and non‐invasive tests performed was also recorded. For the patients' management, we collected data on if they were offered follow‐up clinics, discharged to the care of their GP, re‐referred from their GP to secondary care. Data was collected on specific pharmacological treatment offered, such as first line (antispasmodics, laxatives, antidiarrheals), second line (ondansetron, Enterosgel, linaclotide, prucalopride, rifaximin), and neuromodulators (tricyclic, SSRI or SNRI antidepressants). We noted non‐pharmacological treatments recommended, including BGBT (Gut‐directed hypnotherapy and cognitive behavioral therapy). Data was also collected on dietary aspects of IBS, such as if there was evidence of dietary triggers, first line dietary advice recommended, dietician referral and whether a low FODMAP diet was recommended.

### 2.3 Statistical Analysis

Chi‐squared tests were used to compare nominal data such as their management processes and categorical data with numerical labels such as number of clinicians seen, number of appointments before their diagnosis or number of investigatory tests performed. Mann–Whitney tests were used to compare the median number of days from initial encounter to diagnosis and median invasive and non‐invasive tests.

All statistical analysis was performed using Statistical Package for Social Sciences 30.0.0 (SPSS). Statistical significance was defined by a *p* ≤ 0.05.

## 3 Results

### 3.1 Study Population Characteristics

During the 32‐month period, 136 age and gender matched patients with IBS that met the inclusion criteria were included in this study, with 68 self‐identifying as one of 12 ethnic groups we grouped under an ethnic minority cohort, with the most common being Asian Pakistani (Figure 1 and Table 1).

**TABLE 1:** Demographics of the patient cohort including the number of patients with each IBS Subtype in each ethnic group.

		White British (*n* = 68)	Ethnic minorities (*n* = 68)	*p*
Age (mean)		40.93	40.96	0.99
Gender (number of females)		42 (61.8%)	42 (61.8%)	1.00
IBS subtype	IBS‐C	17 (25%)	18 (26.5%)	0.77
	IBS‐D	26 (38.2%)	22 (32.3%)	
	IBS‐Mixed	25 (36.8%)	28 (41.2%)	

Figures S1 and S2 show the percentage of patients in each religious group for each cohort. The most common religion in the White British cohort is Christianity and in the Ethnic minority cohort 60% of patients were Muslim.

**FIGURE 2:**
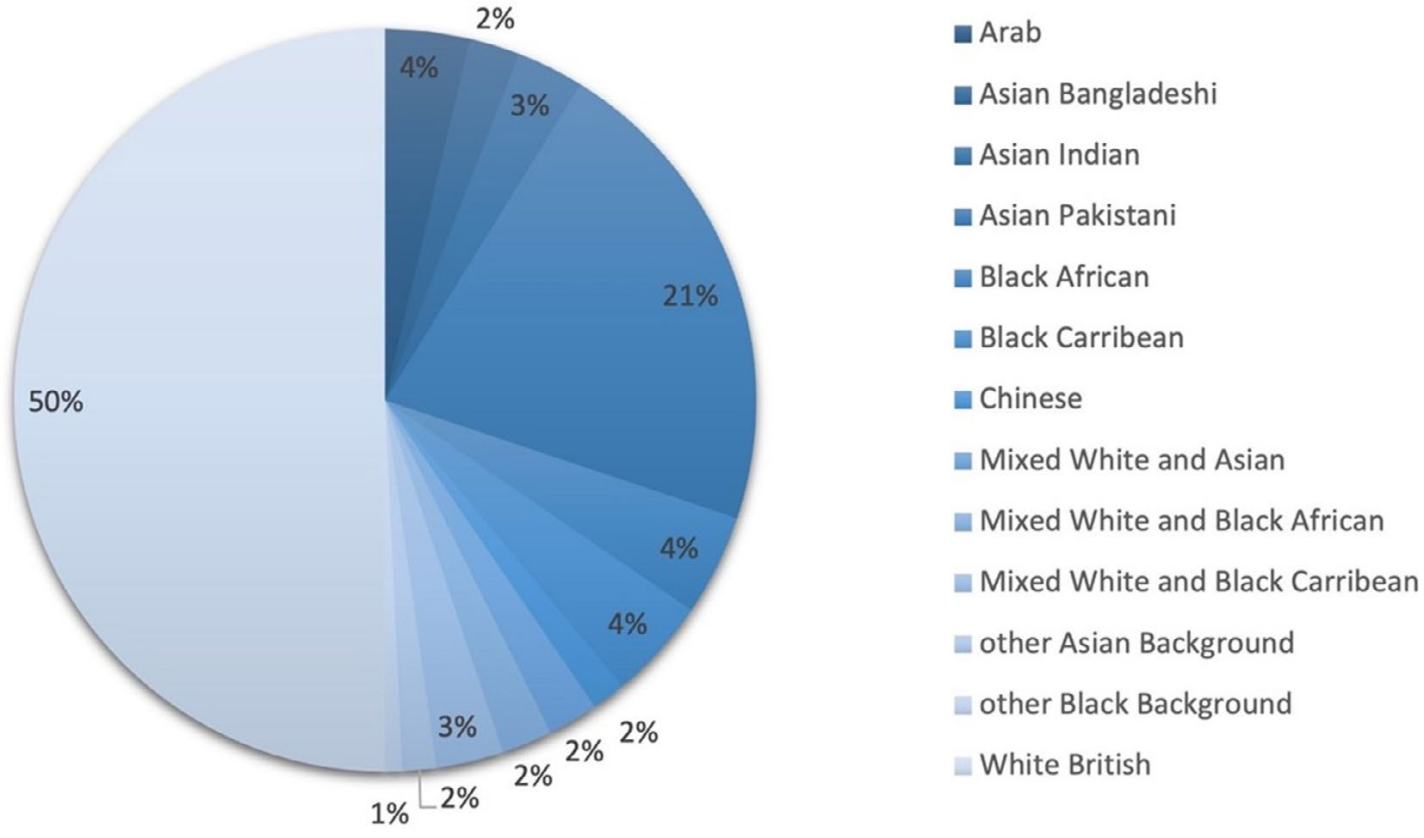
The percentage of patients in each ethnic group.

The proportion of IBS subtypes was similar in both cohorts and the most common subtype across the whole patient population was IBS‐mixed (Figures 2 and S3).

The number of patients that had a diagnosis of a relevant comorbidity in each cohort was recorded and across both cohorts the prevalence of common mental disorders was similar (Figure S4).

### 3.2 Diagnostic Work Up

Overall, the proportion of patients with a diagnosis of IBS in primary care or on initial secondary encounter was low in both groups (Table 2). Of White British patients, 57.4% only saw one clinician in their IBS diagnostic process, compared to only 33.8% of ethnic minorities (*p* = 0.012). Furthermore, 33.8% of ethnic minority patients saw 2 clinicians, and 16.2% saw 3 or more clinicians for their IBS diagnosis, compared to only 20.6% and 4.4% for the White British controls (*p* = 0.012). Additionally, 57.4% White British patients had a single appointment to receive their IBS diagnosis in secondary care, whereas only 35.5% of ethnic minorities had the same (*p* = 0.007). 25.0% of ethnic minority patients needed 3 or more secondary clinical appointments to receive their IBS diagnosis, compared to only 5.9% of White British patients (*p* = 0.007, Table 3).

**TABLE 2:** IBS diagnosis made in primary or secondary care. This table represents the number of patients with a known primary care IBS diagnosis, or those diagnosed at their first secondary care appointment.

	White British *n* = 68	Ethnic minorities *n* = 68	Overall *n* = 136	*P*
Number of patients diagnosed with IBS in primary care	12 (17.6%)	11 (16.2%)	23 (16.9%)	0.819
Number of patients diagnosed with IBS on initial secondary appointment.	23 (33.8%)	17 (25.0%)	40 (29.4%)	0.259

**TABLE 3:** Number of secondary care clinicians and appointments White British and Ethnic Minority patients required to receive their IBS diagnosis.

		White British *n* = 68	Ethnic minorities *n* = 68	Overall *n* = 136	*p*
Number of clinicians involved in secondary care to make the diagnosis of IBS	0	12 (17.6%)	11 (17.6%)	23 (16.9%)	**0.012**
	1	39 (57.4%)	23 (33.8%)	62 (45.6%)	
	2	14 (20.6%)	23 (33.8%)	37 (27.2%)	
	≥ 3	3 (4.4%)	11 (16.2%)	14 (10.3%)	
Number of secondary clinic appointments to make a diagnosis of IBS	0	12 (17.6%)	11 (16.2%)	23 (16.9%)	**0.007**
	1	39 (57.4%)	24 (35.3%)	63 (46.3%)	
	2	13 (19.1%)	16 (23.5%)	29 (21.3%)	
	≥ 3	4 (5.9%)	17 (25.0%)	21 (15.4%)	

The median number of days from initial secondary encounter to diagnosis for patients who didn't have a primary diagnosis was 156 days for White British, but 296 days for ethnic minorities (U = 1223.5, *p* = 0.105, Table 4). The median number of non‐invasive tests performed on white British to investigate their IBS symptoms was 2, compared to a median of 3 for ethnic minority patients (*p* = 0.093). Similarly, the median number of invasive tests performed was 3 for ethnic minority patients, and 2 for White British patients (*p* = 0.156).

**TABLE 4:** The median number of days from first secondary care appointment to receiving an IBS diagnosis in White British and Ethnic Minority patients.

	White British	Ethnic minorities	U value	*P*
Median number of days from initial secondary encounter to IBS diagnosis (excl. those with a primary diagnosis)	156	296	1223.5	0.105
Median number of non‐invasive investigations a patient had	2	3	1935.0	0.093
Median number of invasive investigations a patient had	2	3	1992.5	0.156

Table S1 demonstrate that both cohorts were investigated similarly during the diagnostic workup. Fecal calprotectin was checked appropriately in line with BSG guidelines in most patients with IBS‐D across the study (37/48 patients with IBS‐D, 77.1%), with no difference between the ethnic groups (*p* = 0.159). Across both cohorts, 30.9% patients did not have a coeliac serology checked, whilst the majority (73.5%) had at least one colonoscopy. Patients that had a colonoscopy in both ethnic groups were older (mean 43.2 ± 15.7 vs. 32.8 ± 13.1 years old, U = 1061, *p* < 0.001), than those that did not have a colonoscopy. The diagnostic investigations were similar in both ethnic groups, with the only exception that Ethnic minority patients with IBS were more likely to have abdominal ultrasonography and White British patients were more likely to have colonic biopsies than their ethnic minority counterparts. However, a sub‐analysis comparing the proportion of patients that had colonic biopsies for microscopic colitis appropriately in the context of IBS‐D only, did not show a significant difference between the ethnic groups (*p* = 0.371).

### 3.3 Comparing the Management of IBS Between Ethnic Groups

Most White British (80.9%) and ethnic minority patients with IBS (69.1%) were offered a follow‐up appointment after being diagnosed. Similarly, most White British (91.2%) and ethnic minority patients with IBS (80.9%) were given symptomatic treatment recommendations. Evidence of first line and neuromodulator treatments offered was similar across both cohorts; however, a significantly greater proportion of White British patients (27.9%) had been offered second line treatment for their IBS, whereas only 8.8% of ethnic minorities were offered the same (*p* = 0.004). The proportion of patients that were noted to have evidence of dietary triggers and received first line dietary recommendations for IBS was similar between ethnic groups, along with similar clinical practice for secondary dietary treatment such as low FODMAP diets and dietician referrals. In the White British cohort, 17.6% were recommended for Brain‐Gut Behavioral therapies (gut‐directed hypnotherapy or CBT), compared to only 2.9% of ethnic minorities (*p* = 0.005). Overall, 59 (43.4%) of patients were discharged back to their GP after their IBS diagnosis, with no difference in discharge rates between ethnic groups (*p* = 0.60, Table 4). However, of those discharged, 32/59 (54.2%) were re‐referred to secondary care due to ongoing symptoms. Re‐referral rates among those with IBS that were discharged were similarly high in both ethnic groups (19/31, 61.2% ethnic minorities vs. 13/28, 46.4% White British, *p* = 0.25).

## 4 Discussion

In our retrospective service evaluation of 136 patients with IBS, we found disparity in diagnosis and management of IBS between ethnic minority patients compared to the White British control group. Consistent with our hypothesis, ethnic minority patients had more secondary clinical appointments before their diagnosis of IBS compared to the White British patients. Furthermore, they had to see more clinicians during their IBS diagnostic process, with half the patients having to see 2 or more different clinicians before receiving their diagnosis (Table 3). On average, ethnic minority patients had to wait longer for their diagnosis and had more investigatory tests (Table 5). However, reassuringly, there was no statistical significance with the number of invasive procedures such as colonoscopies or upper gastrointestinal endoscopies between the two cohorts as seen in previous studies Table S1 from North America [9].

**TABLE 5:** Summary of IBS management in secondary care received by ethnic groups including first‐ and second‐line treatments.

		White British *n* = 68	Ethnic minorities *n* = 68	Overall *n* = 136	*p*
Further clinical follow up after IBS diagnosis?	no	13 (19.1%)	21 (30.9%)	34 (25.0%)	0.113
	yes	55 (80.9%)	47 (69.1%)	102 (75.0%)	
Discharged back to care of GP after diagnosis?	no	40 (58.8%)	37 (54.4%)	77 (56.6%)	0.604
	yes	28 (41.2%)	31 (45.6%)	59 (43.4%)	
Re‐referred to secondary care after being discharged to GP?	no	55 (80.9%)	49 (72.1%)	104 (76.5%)	0.225
	yes	13 (19.1%)	19 (27.9%)	32 (23.5%)	
Symptomatic treatment recommendations made?	no	6 (8.8%)	13 (19.1%)	19 (14.0%)	0.083
	yes	62 (91.2%)	55 (80.9%)	117 (86.0%)	
Evidence of First Line treatments offered? E.g., antispasmodics, laxatives, antidiarrheals	no	13 (19.1%)	15 (22.1%)	28 (20.6%)	0.671
	yes	55 (80.9%)	53 (77.9%)	108 (79.4%)	
Evidence of Second Line treatments offered? E.g., Linaclotide or ondansetron	no	49 (72.1%)	62 (91.2%)	111 (81.6%)	**0.004**
	yes	19 (27.9%)	6 (8.8%)	25 (18.4%)	
Evidence of Neuromodulator treatments offered? E.g., TCAs, SSRIs, SNRIs	no	47 (69.1%)	48 (70.6%)	95 (69.9%)	0.852
	yes	21 (30.9%)	20 (29.4%)	41 (30.1%)	
Evidence of dietary Triggers to IBS flare ups?	no	36 (52.9%)	38 (55.9%)	74 (54.4%)	0.731
	yes	32 (47.1%)	30 (44.1%)	62 (45.6%)	
Evidence of first line dietary IBS recommendations provided?	no	29 (42.5%)	24 (35.3%)	53 (39.0%)	0.379
	yes	39 (57.4%)	44 (64.7%)	83 (61.0%)	
Evidence of dietician referral?	no	39 (57.4%)	44 (67.4%)	83 (61.0%)	0.379
	yes	29 (42.6%)	24 (35.3%)	53 (39.0%)	

These clinically significant differences in the diagnostic work up of the ethnic minority patients mirror previous racial disparities seen in GI populations [5]. Whether this is due to the patient's rejection of the diagnosis of a DGBI or a clinician's apprehension to give such a diagnosis to an ethnic minority patient is unclear. The culture of the clinician, patient and the clinical practice all influence the healthcare interaction and can cause communication difficulties which have been shown to negatively impact health outcomes [12]. Some clinicians may be hesitant to ask about psychosocial factors influencing a patient's IBS symptoms out of concern that it could lead to an extensive discussion of mental health issues or sidetrack the appointment [13]. A patient's history is vital for a confident IBS diagnosis, so if a clinician feels unprepared and inexperienced to confidently extrapolate relevant information from a history, a diagnosis and therefore treatment could be delayed [8, 10].

As observed in our study and others, many ethnic minority patients have a diagnosed common mental health comorbidity such as anxiety and depression. This may be linked to perceived stigma associated with an IBS diagnosis, potentially contributing to a longer diagnostic process and increased use of healthcare resources to establish the diagnosis.

Our data suggest that fewer Ethnic minority patients had been given symptomatic treatment recommendations in secondary care than the White British controls. Furthermore, our data show there was a trend towards ethnic minority patients having more re‐referrals, which could suggest they did not feel their concerns were fully acknowledged or addressed in their initial appointments, causing them to seek secondary care services again. These patterns may reflect disparities in how care is transitioned between primary and secondary providers and could suggest a patient was prematurely discharged without adequate symptomatic relief, or a perception that their condition was less severe. This perception could be influenced by an implicit bias or communication barriers during an appointment [14].

Reassuringly, most ethnic minority and white British patients received first line treatments such as antispasmodics, laxatives and antidiarrheals. However, far fewer ethnic minority patients were offered second line treatments such as Linaclotide or ondansetron compared to the White British cohort. Understanding the biopsychosocial basis of IBS is important for a comprehensive assessment and critical for an effective IBS treatment plan [15]. Therefore, if this difference reflects the clinicians' recommendations, rather than patient related factors, this may indicate either a lack of clinician confidence in prescribing these medications or a potential racial disparity in the application of the IBS treatment guidelines [8].

Notably, over half of both cohorts received dietary advice or a dietician's referral; however, fewer trialed a low FODMAP diet. The low FODMAP diet has a suggested efficacy of 70%; however, applicability of the diet in ethnic minority, specifically Asian populations, is questioned as the culture of food and its ingredients vary so greatly from a typical western diet [16]. It is shown that diet, which is heavily embedded in culture, influences IBS, therefore understanding the role of food in a wide variety of cultures is increasingly important when looking to treat IBS with dietary modifications [1].

Despite evidence that a British Asian population responds well to gut directed hypnotherapy and availability of this treatment at the local tertiary referral centre, our data shows a significantly lower referral of 3% to gut directed hypnotherapy or cognitive behavioural therapy among Ethnic Minority patients compared to 18% of White British patients [17]. This disparity is concerning given the potential effectiveness of this treatment, indicating several factors may contribute to the difference. Clinicians may be less likely to recommend BGBT to Ethnic Minority patients due to implicit bias or the assumption that the patient may not accept that as a treatment option. Additionally, structural barriers such as limited access to culturally adapted services and language barriers may reduce referral rates.

Whilst not the main objective of our paper, our study does suggest that regardless of ethnicity, IBS is not currently being diagnosed and managed in line with BSG guidelines in secondary care. Across the four hospital sites, in a 36‐month period, even allowing for exclusion of patients with IBS that were diagnosed by a tertiary provider, and allowing for possible coding errors, 486 patients diagnosed with IBS in secondary care is a lower‐than‐expected number. Our data suggest that secondary care clinicians lack confidence in making a positive IBS diagnosis based on clinical criteria, and it is possible that a diagnosis of IBS is not being made at all in some patients with IBS. This is reflected in the prolonged average lengths of time in making an IBS diagnosis and despite evidence revealing the yield of colonoscopies in IBS patients being extremely low [18], that nearly three quarters of patients had at least one colonoscopy, both of which contradict guidelines. Some patients have been sent for colonoscopies after a diagnosis of IBS in primary care and with no further outpatient follow‐up. Unnecessary colonoscopies are not only a risk for patients due to its invasive nature, but also a high cost to the NHS, and without clear evidence of its diagnostic value, its role in IBS remains uncertain [8, 18, 19]. Also of concern is the inconsistent approach to non‐invasive testing to exclude IBS mimics. The use of fecal calprotectin was reassuringly appropriate and in line with BSG guidelines, with most patients with IBS‐D across the study having had this non‐invasive test checked, with no difference between the ethnic groups. However, whilst the utilization of coeliac testing was similar between the ethnic groups, 30.9% patients overall had no evidence of a coeliac serology test despite being referred to secondary care for their IBS symptoms. Coeliac disease is four times more likely to be found in those with IBS symptoms than without [20], and therefore we cannot eliminate that some patients in this study may have had undiagnosed coeliac disease.

In terms of IBS management, nearly 40% of patients from both cohorts had no evidence that first line dietary recommendations were given on their records. This figure may be due to a lack of documentation from clinicians on the advice given in appointments or, more concerningly, a lack of adherence to treatment guidelines and the communication of these to patients. Furthermore, only a third of all patients received a gut‐brain neuromodulator for their IBS, despite recent evidence suggesting a low dose of amitriptyline shows significant benefit [20]. Adherence to BSG guidelines for IBS is therefore an important area for future research and national audits and could improve patient outcomes and standardize care practices.

Evidence reveals the burden of IBS on healthcare systems is high, with increasing investigation and treatments options, costing billions of pounds each year in the United Kingdome alone [19]. Despite this, our data suggests that the inefficiency of the IBS care pathway is prevalent. Our data has shown that over half of the patients that were discharged with an IBS diagnosis during the study were referred back to secondary care, suggesting that this cohort of patients was undertreated. Almost two thirds of the patients from the ethnic minority IBS group that were discharged ended up being referred back to secondary care. This highlights the wasteful use of resources in the IBS care pathway that may be due to the lack of progression to second‐line treatments, resulting in potentially preventable further healthcare costs. Future research should focus on identifying modifiable factors in this pathway, such as improving clinicians' communication of a DGBI or improving their confidence in IBS management, to reduce unnecessary use of NHS services.

One key strength of the study is the minimisation of bias, as data was collected using the Slicerdicer tool, without prior knowledge of the patients, ensuring objective and impartial findings. Additionally, the study provides an extensive review of the diagnostic and management processes used for IBS. This thorough analysis, considering 22 diagnostic factors and 12 management steps, enhanced the validity of the study, allowing us to confidently draw conclusions about healthcare utilization.

Several limitations of this study must be acknowledged, as they may influence the interpretation of the findings. A major limitation to our study is the sample size. Although we reviewed all the ethnic minority patients identified by the Slicerdicer tool in the given time frame, this number was limited for several reasons. We focused our study on the treatment of patients in secondary care, and due to the perceived milder nature of IBS, it is possible that a large proportion of ethnic minority patients don't get secondary care referrals for their IBS [9]. Furthermore, due to the nature of our retrospective study we were limited to collecting data based on clinicians' notes and letters during the time the system was used, or scanned copies from previous years uploaded to the platform. This limited us to the assumption that the clinicians' notes encapsulated the entire clinic appointment, including the symptom severity, communication about the diagnosis and any recommendations made. It was also not possible to reliably determine retrospectively whether English was the patients' first language, and whether or not a translator was used for the consultations.

We used the Slicerdicer tool to minimize bias and ensure the inclusion of all patients meeting our criteria, however the tool depends on the accuracy of the clinicians diagnostic coding during patient encounters. A final limitation is that the White British patients were matched from a larger group of > 300 patients, and despite best efforts with matching appropriately, we cannot exclude that the control group were entirely representative of the whole cohort.

Further research should aim to explore the underlying causes of the disparities identified in our study among ethnic minority patients regarding their IBS diagnosis and management.

Given the multifactorial nature of healthcare disparities, identifying a single cause is difficult; therefore, investigations into the influence of cultural perceptions, barriers to communication, and stigma towards a DGBI in different ethnic groups are vital. Studies with larger sample sizes would be vital to assess whether the trends seen in our study, such as delayed diagnosis and overuse of investigatory procedures, are seen at scale.

Finally, enhanced training, aimed at improving clinicians' communication of a DGBI and their confidence in diagnosing and treating IBS in patients from all ethnic backgrounds, is vital to improving treatment equity and reducing the broader strain on NHS resources.

## 5 Conclusion

The findings of our study have shown that disparities exist in the diagnostic and management processes of IBS for ethnic minority patients compared to White British patients. These disparities, whether influenced by cultural, interpersonal, or structural biases and barriers, reveal the necessity for a revised approach to the management of IBS among all ethnicities. Future research should investigate whether these trends are seen on a larger scale, and in other countries with multiethnic populations, whilst investigating the barriers to optimized IBS care for all patients, irrespective of ethnicity or culture.

## Author Contributions

A.N.B., was involved with the study design, data collection, statistical analysis and wrote the initial draft; E.F., helped with study design, data collection and reviewed the manuscript; D.H.V., conceptualized the study, helped with data analysis and interpretation, and helped write and revise the manuscript and is the guarantor.

## Funding

The authors have nothing to report.

## Ethics Statement

Formal ethical approval was not required for this service evaluation. This was confirmed using the University of Manchester Research Ethics Tool and the HRA research ethics decision tool.

## Conflicts of Interest

The authors declare no conflicts of interest.

## Supporting information


**Figure S1:** nmo70272‐sup‐0001‐Supinfo.docx. **Percentage of patients in each religion in the Ethnic minority IBS cohort**.
**Figure S2:** Religious affiliations of the White British IBS cohort.
**Figure S3:** The proportion of IBS subtypes by ethnic group (p = 0.767).
**Figure S4:** The number of patients diagnosed with other relevant comorbidities to IBS by ethnic group.
**Table S1:** The utility of non‐invasive investigations in White British and Ethnic Minority patients with IBS.

## Data Availability

Data can be made available upon reasonable request to the corresponding author.
