# Supplementary material for: Racial Disparities in the Diagnosis and Management Between Secondary Care Ethnic Minority and White British Patients With Irritable Bowel Syndrome
Source: Neurogastroenterol Motil. 2026 Mar 10;38(3):e70272. doi: 10.1111/nmo.70272 (PMC12976173; doi:10.1111/nmo.70272)
Supplement: Supplementary file 1 — Figure S1: nmo70272‐sup‐0001‐Supinfo.docx. Percentage of patients in each religion in the Ethnic minority IBS cohort. Figure S2: Religious affiliations of the White British IBS cohort. Figure S3: The proportion of IBS subtypes by ethnic group (p = 0.767). Figure S4: The number of patients diagnosed with other relevant comorbidities to IBS by ethnic group. Table S1: The utility of non‐invasive investigations in White British and Ethnic Minority patients with IBS. [file NMO-38-e70272-s001.docx]

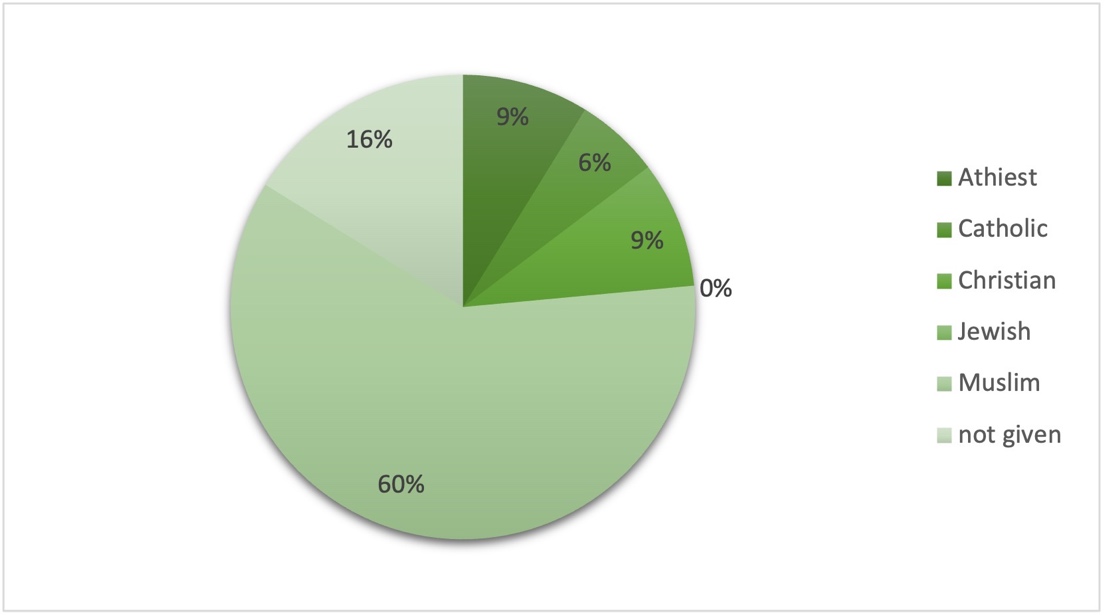


**Supplementary Figure 1: Percentage of patients in each religion in the Ethnic minority IBS cohort.**


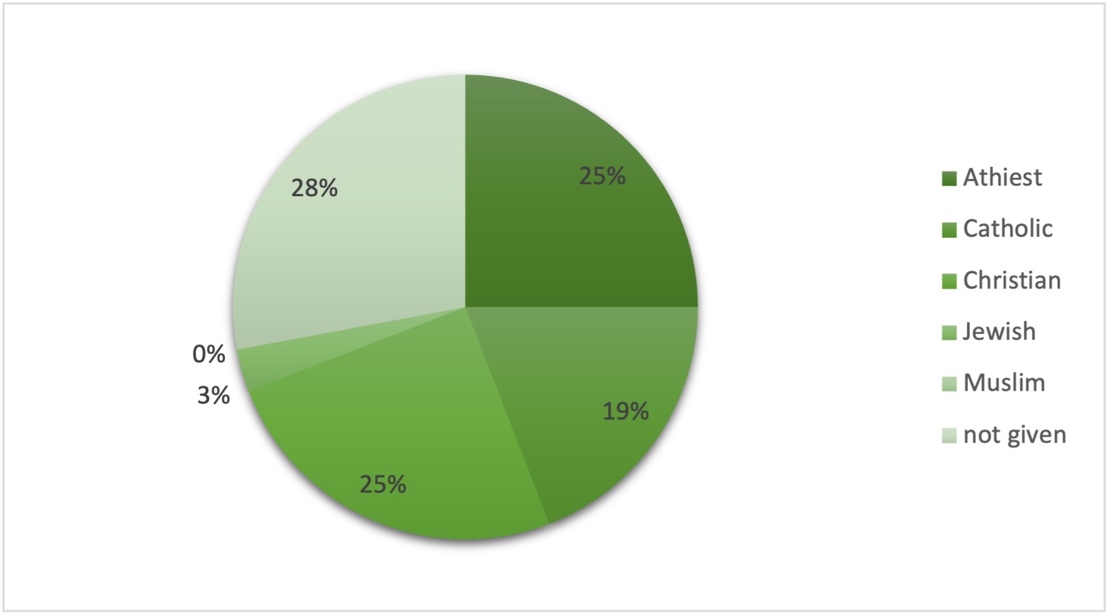


**Supplementary Figure 2: Religious affiliations of the White British IBS cohort.**


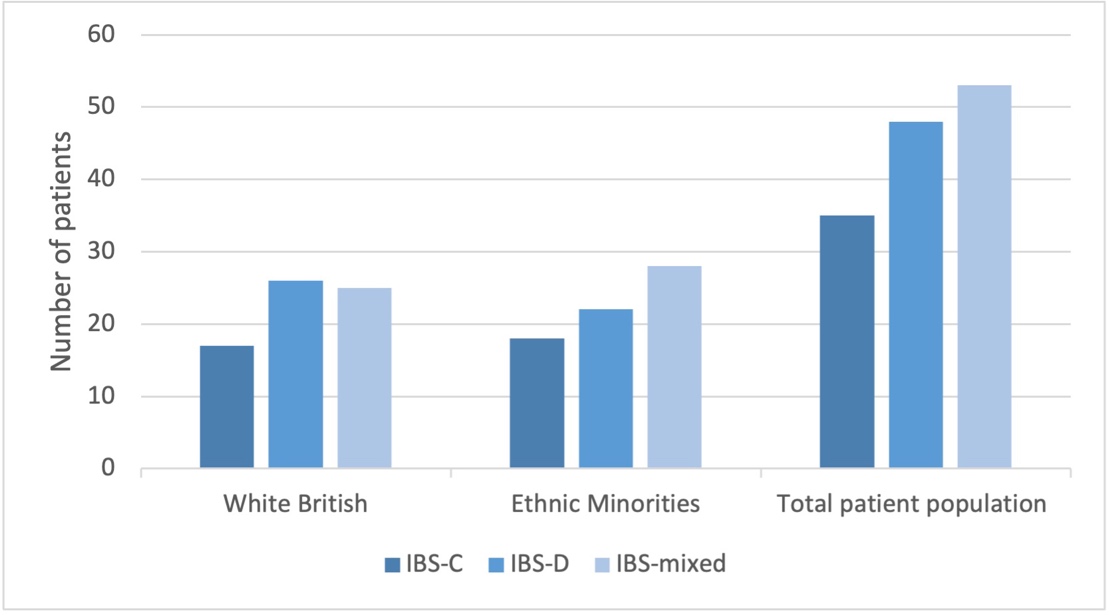


**Supplementary Figure 3: The proportion of IBS subtypes by ethnic group (p=0.767).**


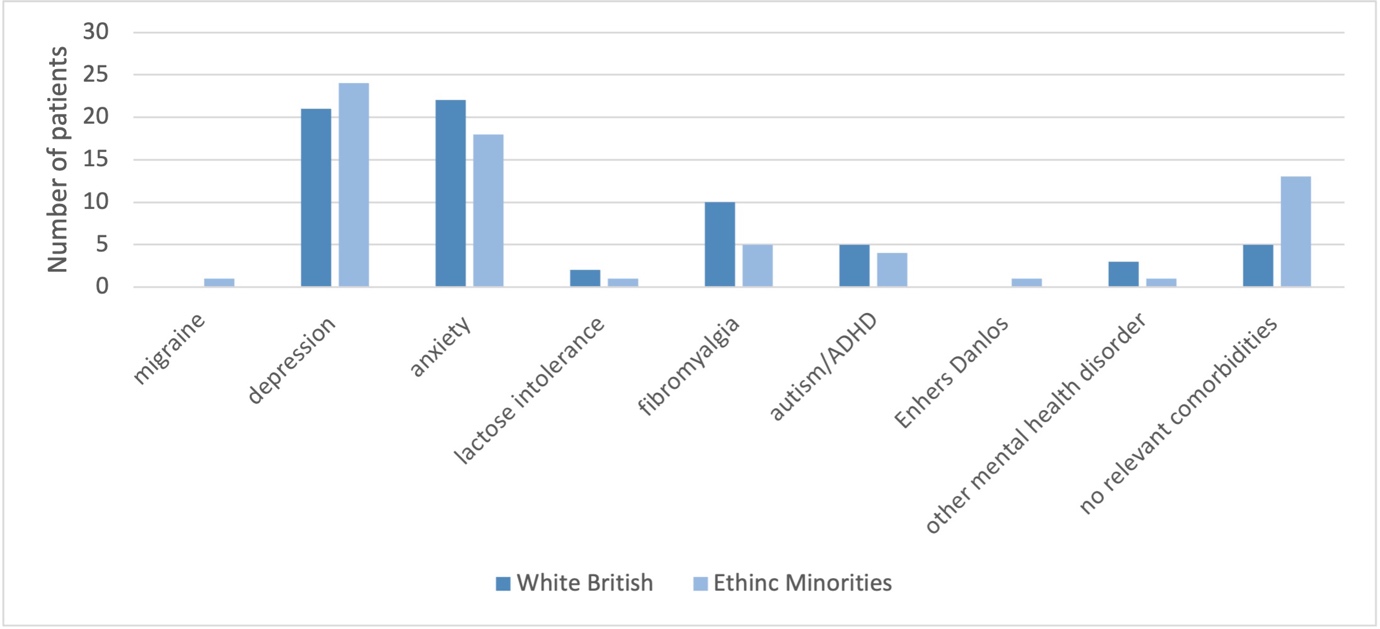


**Supplementary Figure 4: The number of patients diagnosed with other relevant comorbidities to IBS by ethnic group.**

**Supplementary Table 1: The utility of non-****invasive investigations in White British and Ethnic Minority patients with IBS**

**Supplementary Table 2: The utility of invasive investigations in White British and Ethnic Minority patients with IBS**
